# Supplementary material for: Evidence-Based Dietary Practices to Improve Osteoarthritis Symptoms: An Umbrella Review
Source: Nutrients. 2023 Jul 6;15(13):3050. doi: 10.3390/nu15133050 (PMC10347206; doi:10.3390/nu15133050)
Supplement: Supplementary file 1 [file nutrients-15-03050-s001.zip › nutrients-2430989-supplementary.pdf]

Table S1. Search Strategies for 5 Online Databases: PubMed (S1A), CINHAL (S1B), Web of Science (S1C), Cochrane Library (S1D), and Embase (S1E).

S1A. PubMed (MESH Terms)

**Concept**	**Search Terms**
Concept 1: Diet	Food OR "Food"[Mesh] OR "Diet"[Mesh] OR "Diet, Food, and Nutrition/therapeutic use"[Mesh] OR "Diet, Food, and Nutrition"[Mesh] OR "Diet, Carbohydrate-Restricted"[Mesh] OR "Diet, Mediterranean"[Mesh] OR "Mediterranean diet" OR "low carb*" OR "plant based" OR "Diet, Vegetarian"[Mesh] OR "whole grain" OR "Whole Grains"[Mesh] OR "fiber rich" OR nutrition OR diet OR fruit OR "Fruit"[Mesh] OR legumes OR "olive oil" OR "Olive Oil"[Mesh] OR "fish oils" OR "Fish Oils"[Mesh] OR nut OR nuts OR "Nuts"[Mesh] OR vegetable* OR "Vegetables"[Mesh] OR "green tea" OR "Tea"[Mesh] OR turmeric OR "Turmeric extract" [Supplementary Concept] OR ginger OR "Ginger"[Mesh] OR garlic OR spices OR "Spices"[Mesh] OR herbs OR sugar OR "Sugars"[Mesh] OR "red meat" OR fish
Concept 2: Arthritis	Osteoarthritis OR "Osteoarthritis"[Mesh] OR "Osteoarthritis, Knee"[Mesh] OR "Osteoarthritis, Hip"[Mesh] OR "degenerative joint disease" OR "degenerative arthritis" OR arthritis
Concept 3: Outcomes/Measures	"Anti-Inflammatory Agents"[Mesh] OR "Anti-Inflammatory Agents"[Mesh:NoExp] OR "Anti-Inflammatory Agents, Non-Steroidal"[Mesh] OR "Anti-Inflammatory Agents, Non-Steroidal"[Mesh:NoExp] OR "Inflammation"[Mesh] OR "Inflammation Mediators"[Mesh] OR "Inflammation Mediators/blood"[Mesh] OR "Inflammation Mediators/urine"[Mesh] OR "Pain Management"[Mesh] OR "Pain-Free" [Supplementary Concept] OR "Pain"[Mesh] OR "Acute Pain"[Mesh] OR "Musculoskeletal Pain"[Mesh] OR "Chronic Pain"[Mesh] OR "Arthralgia"[Mesh] OR "Mobility Limitation"[Mesh] OR "Range of Motion, Articular"[Mesh]
**Total Results (N)**	3,394
**Filters Applied**	Meta-Analysis, Systematic Review, Species-Human
**Results After Filters Applied (n)**	108

S1B. CINAHL (CINAHL headings)

**Concept**	**Search Terms**
Concept 1: Diet	Food OR (MH "Food") OR (MH "Diet") OR (MH "Diet Therapy") OR (MH "Diet, Low Carbohydrate") OR (MH "Mediterranean Diet") OR "Mediterranean diet" OR "low carb*" OR "plant bas*" OR (MH "Plant-Based Diet") OR (MH "Vegetarianism") OR "whole grain" OR "fiber rich" OR nutrition OR diet OR fruit OR (MH "Fruit") OR legumes OR "olive oil*" OR (MH "Olive Oil") OR "fish oils" OR (MH "Fish Oils")

	OR nut OR nuts OR "(MH Nuts") OR vegetable* OR (MH "Vegetables") OR "green tea" OR (MH "Tea") OR turmeric OR (MH "Turmeric") OR ginger OR (MH "Ginger") OR garlic OR spices OR (MH "Spices") OR herbs OR sugar* OR "red meat" OR fish
Concept 2: Arthritis	Osteoarthritis OR (MH "Osteoarthritis") OR (MH "Osteoarthritis, Knee") OR (MH "Osteoarthritis, Hip") OR "degenerative joint disease" OR "degenerative arthritis" OR arthritis
Concept 3: Outcomes/Measures	(MH "Anti-Inflammatory Agents") OR (MH "Anti-Inflammatory Agents, Non-Steroidal") OR (MH "Inflammation") OR (MH "Inflammation Mediators") OR (MH "Pain Management") OR (MH "Pain") OR (MH "Acute Pain (Saba CCC)") OR (MH "Chronic Pain") OR (MH "Arthralgia")
**Total Results (N)**	811
**Filters Applied**	"systematic review" or "meta-analysis"
**Results After Filters Applied (n)**	40

S1C. Web of Science (10 year rolling back)

Concept	Search Terms
Concept 1: Diet	Food OR "Mediterranean diet" OR "low carb*" OR "plant based" OR "whole grain" OR "fiber rich" OR nutrition OR diet OR fruit OR legumes OR "olive oil" OR "fish oils" OR nuts OR vegetable* OR "green tea" OR turmeric OR ginger OR garlic OR spices OR herbs OR sugar OR "red meat" OR fish
Concept 2: Arthritis	Osteoarthritis OR "degenerative joint disease" OR "degenerative arthritis" OR arthritis
Concept 3: Outcomes/Measures	"anti-inflammatory" OR "non-steroidal" OR inflammation OR "pain management" OR "pain-free" OR pain OR "acute pain" OR "musculoskeletal pain" OR "chronic pain" OR "arthralgia" OR (mobility limitations) OR "range of motion"
**Total Results (N)**	5,323
**Filters Applied**	"systematic review" or "meta-analysis"
**Results After Filters Applied (n)**	229

S1D. Cochrane Library (MESH Terms)

Concept	Search Terms
Concept 1: Diet	Food OR [mh Food] OR [mh Diet] OR "Mediterranean diet" OR "low carb*" OR "plant based" OR [mh "Diet, Vegetarian"] OR "whole grain" OR [mh "Whole Grains"] OR "fiber rich" OR nutrition OR diet OR fruit OR [mh Fruit] OR legumes OR "olive oil" OR [mh "Olive Oil"] OR "fish oils" OR [mh "Fish Oils"] OR nut OR nuts OR [mh Nuts] OR vegetable* OR [mh Vegetables] OR "green tea" OR [mh Tea] OR

	turmeric OR [mh “Turmeric extract"] OR ginger OR [mh Ginger] OR garlic OR spices OR [mh Spices] OR herbs OR sugar OR [mh Sugars] OR “red meat” OR fish
Concept 2: Arthritis	Osteoarthritis OR [mh Osteoarthritis] OR “degenerative joint disease” OR “degenerative arthritis” OR arthritis
Concept 3: Outcomes/Measures	[mh "Anti-Inflammatory Agents"] OR [mh Inflammation] OR [mh "Inflammation Mediators"] OR [mh "Pain Management"] OR [mh "Pain-Free"] OR [mh Pain] OR [mh "Acute Pain"] OR [mh "Musculoskeletal Pain"] OR [mh "Chronic Pain"] OR [mh Arthralgia] OR [mh "Mobility Limitation"]
**Total Results (N)**	400
**Filters Applied**	"systematic review" or "meta-analysis"
**Results After Filters Applied (n)**	97

S1E. Embase

**Concept**	**Search Terms**
Concept 1: Diet	(Food OR “Mediterranean diet” OR “low carb*” OR “plant based” OR “whole grain” OR “fiber rich” OR nutrition OR diet OR fruit OR legumes OR “olive oil” OR “fish oils” OR nuts OR vegetable* OR “green tea” OR turmeric OR ginger OR garlic OR spices OR herbs OR sugar OR “red meat” OR fish)
Concept 2: Arthritis	(Osteoarthritis OR “degenerative joint disease” OR “degenerative arthritis” OR arthritis)
Concept 3: Outcomes/Measures	(“anti-inflammatory” OR “non-steroidal” OR inflammation OR “pain management” OR “pain-free” OR pain OR “acute pain” OR “musculoskeletal pain” OR "chronic pain” OR "arthralgia" OR (mobility limitations) OR “range of motion”)
**Filters Applied**	Humans, Systematic Review, Meta Analysis
**Entire Search Statement**	(food OR 'mediterranean diet' OR 'low carb*' OR 'plant based' OR 'whole grain' OR 'fiber rich' OR nutrition OR diet OR fruit OR legumes OR 'olive oil' OR 'fish oils' OR nuts OR vegetable* OR 'green tea' OR turmeric OR ginger OR garlic OR spices OR herbs OR sugar OR 'red meat' OR fish) AND (osteoarthritis OR 'degenerative joint disease' OR 'degenerative arthritis' OR arthritis) AND ('anti-inflammatory' OR 'non-steroidal' OR inflammation OR 'pain management' OR 'pain-free' OR pain OR 'acute pain' OR 'musculoskeletal pain' OR 'chronic pain' OR 'arthralgia' OR (mobility AND limitations) OR 'range of motion') AND ([systematic review]/lim OR [meta analysis]/lim) AND [humans]/lim AND [embase]/lim
**Total Results (N)**	499
